# Supplementary material for: Synthesis, Characterization and Visible-Light Photocatalytic Activity of Solid and TiO2-Supported Uranium Oxycompounds
Source: Nanomaterials (Basel). 2021 Apr 19;11(4):1036. doi: 10.3390/nano11041036 (PMC8073566; doi:10.3390/nano11041036)
Supplement: Supplementary file 1 [file nanomaterials-11-01036-s001.zip › nanomaterials-1171395-supplementary.pdf]

Synthesis, characterization and visible-light photocatalytic activity of solid and TiO_2-supported uranium oxycompounds

Mikhail Lyulyukin, Tikhon Filippov, Svetlana Cherepanova, Maria Solovyeva, Igor Prosvirin, Andrey Bukhtiyarov, Denis Kozlov, Dmitry Selishchev

Boreskov Institute of Catalysis, Novosibirsk 630090, Russian Federation

Supporting Information

XRD analysis

The phase composition was analyzed by X-ray powder diffraction using a D8 Advance diffractometer (Bruker, USA) equipped with a CuKα radiation source and a LynxEye position sensitive detector. Fig. S1 shows XRD patterns for the sample prepared via hydrothermal treatment and for $UO_{2.12}$ reference (PDF#04-007-2508)

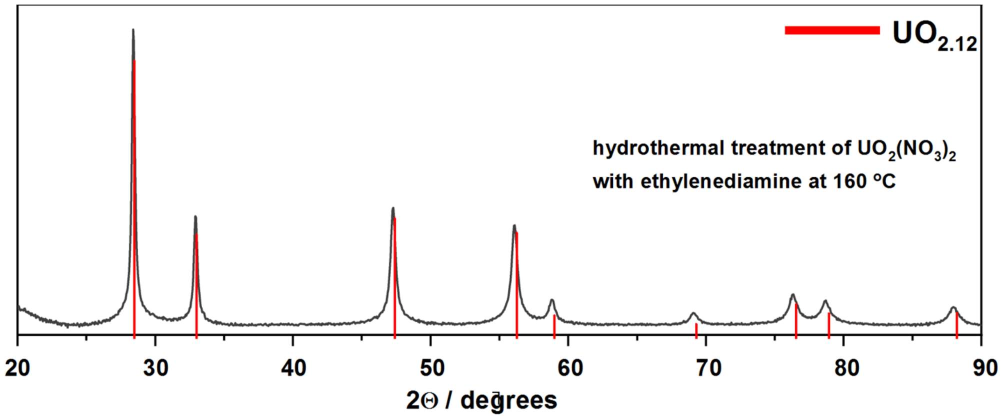

Figure S1. XRD patterns for the sample prepared via hydrothermal treatment and for $UO_{2.12}$ reference (PDF#04-007-2508).

DRIFT analysis

In addition to XPS, diffuse reflectance infrared Fourier transform (DRIFT) spectroscopy was used for the surface characterization of TiO_2-supported uranium compounds. DRIFT spectra

were recorded at room temperature using a Vector 22 spectrometer (Bruker, USA) equipped with a diffuse reflectance accessory from PIKE Technologies (USA). The studied range was 800–4000 cm^{-1} with a resolution of 1 cm^{-1}.

As the **$UO_2(NO_3)_2/TiO_2$-T500** sample is **$UO_2(NO_3)_2/TiO_2$** ones after heating at 500 °C, the expected changes are in decreasing of the specific surface area and the removal of the surface nitrate groups. DRIFT spectroscopy was employed to visualize the last one. Fig. S2 shows the spectra for **$UO_2(NO_3)_2/TiO_2$** and **$UO_2(NO_3)_2/TiO_2$-T500**. The most noticeable differences between these samples are observed in the ranges of wave numbers 650–1050 cm^{-1} and 1150–1550 cm^{-1}. These ranges correspond to the oscillation of uranyl groups and nitro groups, respectively [55].

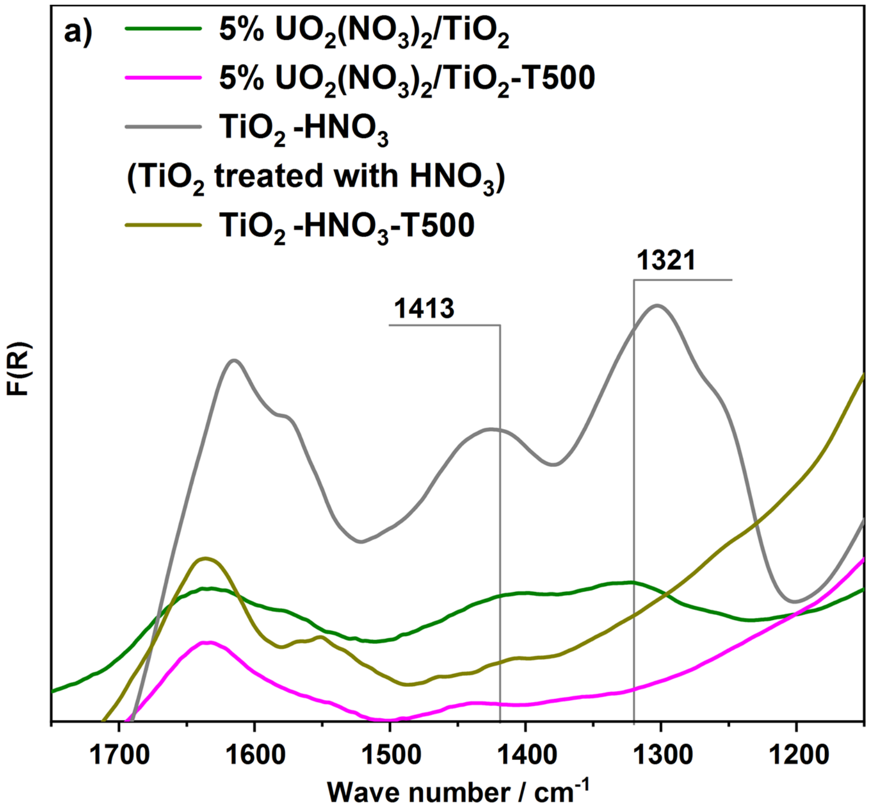

Figure S2. DRIFT spectra of TiO_2-supported materials.

For clear justification of the signal observed, TiO_2 Hombifine N treated with nitric acid (**TiO_2-HNO_3**), and that one after calcination at 500 °C (**TiO_2-HNO_3-500**) were characterized using IR spectroscopy too.

Fig. S2 illustrates the changes in spectra after the immobilization of nitrate groups on the TiO_2 surface: both the **$UO_2(NO_3)_2/TiO_2$** and **TiO_2-HNO_3** have the detectable signals in the ranges of 1380-1450 cm^{-1} and 1260-1330 cm^{-1}, which match well to oscillations $\upsilon(NO_3^-)$ and $\upsilon(-O-NO_2)$, respectively [55]. The position and shape of the peak for the different processes of additives application onto the surface allow matching them with nitro groups. This is in an agreement with their absence after heating of the samples at 500 °C, because the nitro-groups are known to leave the surfaces at high temperatures in the oxygen-containing atmosphere as nitrogen oxides.

Photonic efficiency

Instead of the quantum (or photonic) efficiency of the product formation, many researchers estimate the efficiency for the reaction in whole in an assumption that one light quanta absorbed by (or incident on) the photocatalyst changes the oxidation state of carbon and oxygen into the oxidizing substrate and molecular oxygen by 1. According to the stoichiometry of complete oxidation of acetone:

$$CH_3COCH_3 + 4O_2 = 3CO_2 + 3H_2O$$

the photonic efficiency for this reaction can be estimated as follows:

$$\xi = \frac{\frac{1}{3}\, dn_{CO_2}/dt}{\frac{1}{16} q_p^0} = \frac{16}{3}\xi_{CO_2}$$

where 3 is the stoichiometric coefficient for CO_2, 16 is the number of quanta required for the complete oxidation of an acetone molecule.

Based on the value of 2.3% for ξ_{CO_2} (see the main text of manuscript), the photonic efficiency of acetone oxidation can be estimated as 12.2%. This is a very high value for the complete oxidation of organic compounds in the gas phase under visible light.

Emission spectrum of LED

Highly power light-emitting diode (LED) plate with a special collimating lens was employed for the photocatalyst irradiation during the experiments. LED provided radiation in a range of 405–520 nm with a maximum at λ_{max} = 450 nm (Fig. S3). The irradiance measured using an ILT 950 spectroradiometer from International Light Technologies Inc. (USA) was 93.5 mW cm^{-2}.

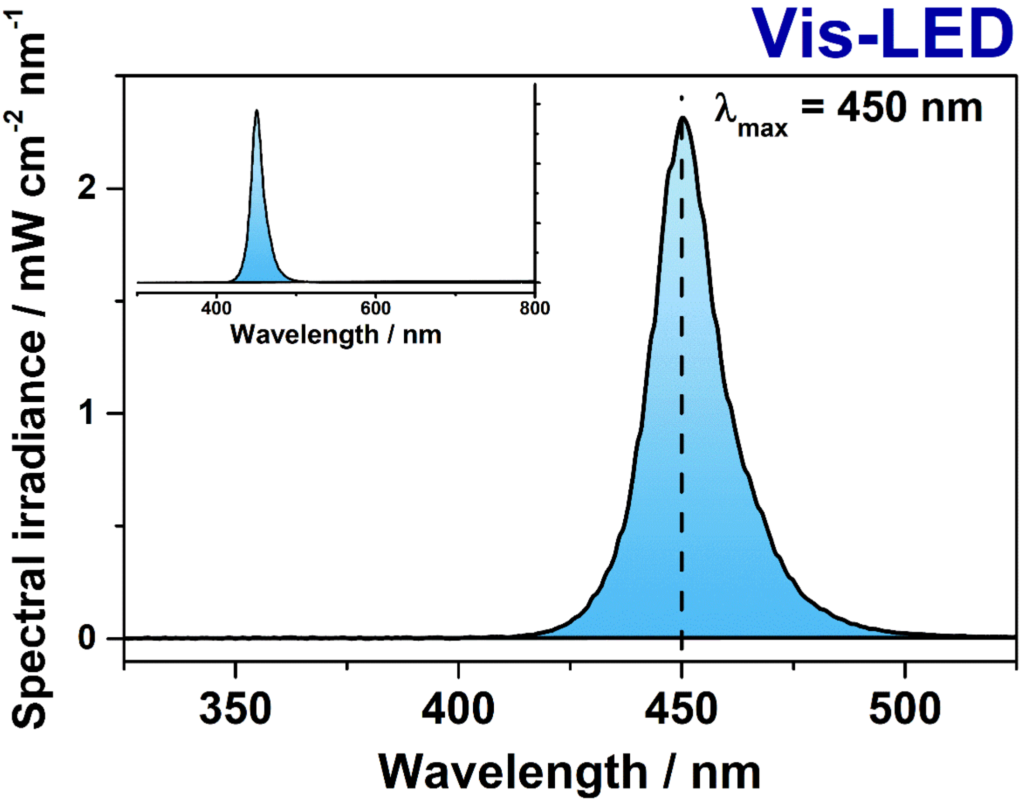

Figure S3. Emission spectrum of used light-emitted diode.

References

[1] J.F. Moulder, W.F. Stickle, P.E. Sobol, K.D. Bomben, Handbook of X-Ray Photoelectron Spectroscopy, Perkin-Elmer Corp, Eden Prairie, Minnesota, USA, 1992.

[2] C.D. Wagner, A.V. Naumkin, A. Kraut-Vass, J.W. Allison, C.J. Powell, C.J. Rumble, NIST X-ray Photoelectron Spectroscopy Database, Version 3.5, Natl. Inst. Stand. Technol. Gaithersbg. (2003).

[3] K. Okada, T. Uozumi, A. Kotani, Charge-Transfer Satellites in Ti2p XAS and XPS of Ti Compounds, Jpn. J. Appl. Phys. 32 (1993) 113–115.

[4] E.L. Bullock, L. Patthey, S.G. Steinemann, Clean and hydroxylated rutile TiO2 (110) surfaces studied by X-ray photoelectron spectroscopy, Surf. Sci. 352–354 (1996) 504–510.
